# Comparison of the Results of Prenatal and Postnatal Echocardiography and Postnatal Cardiac MRI in Children with a Congenital Heart Defect

**DOI:** 10.3390/jcm12103508

**Published:** 2023-05-17

**Authors:** Marios Mamalis, Tamara Koehler, Ivonne Bedei, Aline Wolter, Johanna Schenk, Ellyda Widriani, Roland Axt-Fliedner

**Affiliations:** Division of Prenatal Medicine & Fetal Therapy, Department of Obstetrics & Gynecology, Justus-Liebig-University Giessen, 35390 Giessen, Germany

**Keywords:** congenital heart disease, echocardiography, postnatal cardiac MRI

## Abstract

Objective: In fetuses with suspicion of congenital heart disease (CHD), assessment by segmental fetal echocardiography is of great importance. This study sought to examine the concordance of expert fetal echocardiography and postnatal MRI of the heart at a high-volume paediatric heart centre. Methods: The data of two hundred forty-two fetuses have been gathered under the condition of full pre- and postnatal and the presence of a pre- and postnatal diagnosis of CHD. The haemodynamically leading diagnosis was determined for each test person and was then sorted into diagnostic groups. The diagnoses and diagnostic groups were used for the comparison of diagnostic accuracy in fetal echocardiography. Results: All comparisons between the diagnostic methods for detection of congenital heart disease showed an “almost perfect” (Cohen’s Kappa > 0.9) strength of agreement for the diagnostic groups. The diagnosis made by prenatal echocardiography showed a sensitivity of 90–100%, a specificity and a negative predictive value of 97–100%, and a positive predictive value of 85–100%. The diagnostic congruence resulted in an “almost perfect” strength of agreement for all evaluated diagnoses (transposition of great arteries, double outlet right ventricle, hypoplastic left heart, tetralogy of Fallot, atrioventricular septal defect). An agreement of Cohen’s Kappa > 0.9 was achieved for all groups, with exception of the diagnosis of double outlet right ventricle (0.8) in prenatal echocardiography compared to postnatal echocardiography. This study came to the result of a sensitivity of 88–100%, a specificity and negative predictive value of 97–100%, and a positive predictive value of 84–100%. The performance of cardiac magnetic resonance imaging (MRI) as an additional measure to echocardiography had an added value in the description of the malposition of the great arteries when diagnosed with double outlet right ventricle and in the detailed description of the anatomy of the lung circulation. Conclusions: Prenatal echocardiography could be shown to be a reliable method for detection of congenital heart disease when regarding the slightly lower accuracy of diagnosis for double outlet right ventricle and right heart anomalies. Furthermore, the impact of examiner experience and the consideration of follow-up examinations for further improvement of diagnosis accuracy may not be underestimated. The main advantage of an additional MRI is the possibility to obtain a detailed anatomic description of the blood vessels of the lung and the outflow tract. The conduction of further studies that include false-negative and false-positive cases, and studies that are not set within the high-risk-group, as well as studies in a less specialized setting, would allow the completion and investigation of possible differences and discrepancies when comparing the results that have been obtained in this study.

## 1. Introduction

Congenital heart defects (CHD) represent a group of congenital anomalies difficult to characterize prenatally. For this reason, an antenatally suspected congenital heart defect (CHD) via a fetal echocardiography is postnatally confirmed by a postnatal echocardiography and, on some occasions, postnatal cardiac magnetic resonance imaging (MRI) is adjunctively required.

In this retrospective explorative analysis, we describe our experience in the clinical management of two hundred forty-two fetuses with suspected congenital heart defects (CHD) referred to our unit for diagnosis and management over a five-year period.

The objective of this study is to assess whether and to what extent the prenatal echocardiography is a reliable method for diagnosis of several CHD and if postnatal cardiac MRI provides additional diagnostic value.

## 2. Methods

During a five-year study period from 2012 to 2017, there have been two hundred forty-two cases with suspected CHD referred to our fetal medicine unit (FMU) at the University Hospital of Giessen and Marburg (UKGM) for diagnosis and further management. All individuals, in addition to prenatal echocardiography, have underwent postnatal echocardiography and postnatal cardiac MRI examination, when indicated. Prenatal and postnatal echocardiography have been performed in accordance to published guidelines [1,2,3].

Statistical analysis was performed by the Institute of Biostatistics at Justus-Liebig University Giessen. The interpretation of results has been based on Cohen’s Kappa coefficient and the concordance rate of Landis and Koch. The congenital heart defects’ spectrum that has been met in our patient’s collective is demonstrated in Table 1. The level of significance when testing concordance for all Cohen’ s Kappa scores has been a: 5%. Additionally, the Bonferroni correction method and *p*-value have been utilized. Overall results are presented as descriptive data. Sensitivity (SEN) and specificity (SPE), and positive (PPV) and negative predictive values (NPV) were calculated.

## 3. Results

Two hundred forty-two cases were included in the study. Twenty-four cases were excluded due to termination of pregnancy; seven intrauterine demises occurred. Two hundred eleven cases remained for final analysis.

Table 2 shows the overall prevalence of CHD in our cohort. The strength of agreement for CHD in the different groups has been evaluated (Table 3). Comparing the several CHD in the prenatal-postnatal echocardiography group, the anomalies of right heart and complex CHD have shown the lowest strength of agreement (Cohen’ s Kappa: 0.92). For conotruncal and outflow tract anomalies, and septal defects, the strength of agreement has been 0.93 and for left heart anomalies, 0.96.

The lowest strength of agreement has been indicated for septal defects in the prenatal echocardiography-MRI group (Cohen’s Kappa: 0.91). The anomalies of right heart and complex CHD have shown a concordance of Cohen’s Kappa as high as 0.94 and the conotruncal and outflow tract anomalies’ Cohen’s Kappa has been 0.95. The left heart anomalies have demonstrated the second highest strength of agreement (Cohen’s Kappa: 0.98) below the “Other anomalies” group.

In the postnatal echocardiography-postnatal MRI group, the diagnosis of septal defects has been “perfectly” concordant. The left heart anomalies have shown a concordance rate of 0.98 and the conotruncal and outflow tract anomalies, and right heart anomalies have similarly achieved high concordance (Cohen’s Kappa: 0.97).

The comparison of the precision of the diagnosis via different diagnostic methods by utilized Cohen’s Kappa has proved to have a significance of *p* < 0.001 in all cases. After implying the Bonferroni correction, all tests for CHD have proved significance.

In Table 4, the predictive parameters for CHD via prenatal echocardiography, compared to postnatal echocardiography, are demonstrated; “Other anomalies” have achieved a positive predictive value (PPV) of 100%. Conotruncal and outflow tract anomalies, and right and left heart anomalies have shown a positive predictive value (PPV) higher than 94%. A low PPV score of 88% and 86% could be reported for septal defects and complex CHD, respectively. In all groups, a PPV higher than 97% has been achieved. The lowest sensitivity (SEN) has been shown for right heart anomalies (90%), while the rest of CHD have demonstrated high sensitivity (SEN) and specificity (SPE) scores (Table 5).

In the prenatal echocardiography-MRI group, the right heart anomalies group has shown the lowest sensitivity (SEN) (90%). Prenatal echocardiography has been proved to provide a high accuracy for diagnosis of left heart anomalies, conotruncal and outflow tract anomalies, and “Other anomalies”, as SEN lies as high as 97.5%, 95.8%, and 100%, respectively. Similarly, specificity (SPE) has shown a very high score (>97%) for all CHD. The conotruncal and outflow tract anomalies, septal defects, and complex CHD have reached a specificity (SPE) of 97.9–99%. For left heart anomalies, right heart anomalies, and “Other anomalies”, the SPE has been 100%. Right heart anomalies, despite low SEN, have demonstrated a PPV score of 100%. PPV scores for conotruncal and outflow tract anomalies have been 95.8%, and 100% for left heart anomalies, right heart anomalies, and “Other anomalies”. Septal defects have possessed the lowest PPV score of 85.7%. Negative predictive value (NPV) has reached a high score (>97%). Septal defects, complex CHD, and “Other anomalies” have reached a negative predictive value (NPV) of 100%.

The postnatal echocardiography-MRI group has achieved an “almost perfect” strength of agreement in all CHD. A Cohen’s Kappa score of 1 has been shown for septal defects and “Other anomalies”, 0.98 for conotruncal and outflow tract anomalies, and 0.97 for right heart abnormalities. The lowest rate has been related to complex CHD with Cohen’s Kappa (0.94). For all groups, there is a statistical significance for Cohen’s Kappa before and after the Bonferroni correction. For conotruncal and outflow tract anomalies, and left heart anomalies, an SEN score of 100% and an SPE higher than 98% could be achieved. For right heart anomalies, an SEN of 95.5% for postnatal echocardiography has been shown. The lowest SEN of 89% has been related to complex CHD, and it has represented the only one with a score lower than 95%. A high SPE (98%) has been achieved in all groups. For right heart anomalies, septal defects, complex CHD, and “Other anomalies”, an SPE of 100% has been achieved. For conotruncal and outflow tract anomalies, and left heart anomalies, an SPE higher than 98% has been shown (Table 6). The right heart anomalies, septal defects and “Other anomalies” have achieved a PPV of 100%. Moreover, the conotruncal and outflow tract anomalies, left heart anomalies, septal defects, and “Other anomalies” have shown an NPV as high as 100%. The right heart anomalies with an NPV of 99% indicate that there is 1% chance of a false-negative result.

The concordance of transposition of great arteries (TGA), double outlet right ventricle (DORV), hypoplastic left heart (HLH), tetralogy of Fallot (ToF), and atrioventricular septum defect (AVSD) in the prenatal-postnatal echocardiography group has been “almost perfect” achieving Cohen’s Kappa higher than 0.8 (Table 7). Double outlet right ventricle (DORV) has been the only entity with a Cohen’s Kappa score lower than 0.9. In the prenatal echocardiography-MRI group, double outlet right ventricle (DORV) and atrioventricular septal defect (AVSD) have achieved an “almost perfect” classification with a Cohen’s Kappa score lower than 0.9. In case of transposition of great arteries (TGA), hypoplastic left heart (HLH), and tetralogy of Fallot (ToF), the concordance rate has been 1. A same pattern is used to evaluate the concordance of transposition of great arteries (TGA), double outlet right ventricle (DORV), hypoplastic left heart (HLH), tetralogy of Fallot (ToF), and atrioventricular septal defect (AVSD) in the postnatal echocardiography-MRI group. For a concordance with a score higher than 0.9 for all and specifically for AVSD, a rate of 1 has been shown. For all groups, a statistical significance could be shown, which was consistent after Bonferroni correction.

Table 8 shows the quality criteria SEN and SPE for TGA, DORV, HLH, ToF, and AVSD in the prenatal-postnatal echocardiography group. Diagnosis of DORV has achieved the lowest sensitivity of 88.9%. The rest have achieved an SEN higher than 94%. Particularly, TGA and ToF have been as high as 95%. HLH has shown an SEN of 97%, while AVSD, 100%. All groups have presented an SPE higher than 97%, while TGA and HLH have had an SPE as high as 100%. Similarly, ToF has shown an SPE of 99.4%. The lowest SPE has corresponded to DORV (98.1%) and AVSD (97.4%). The PPV has been 85% for DORV and AVSD, while the PPV was 95% for ToF and 100% for TGA and HLH. The NPV for all CHD has achieved a score higher than 98%. The lowest NPV has corresponded to DORV (98.7%). For the rest, the NPV has been as high as almost 100%.

Table 9 shows that the quality criteria used for the prenatal echocardiography-postnatal MRI group have represented a high overall SPE (>97%); in particular for AVSD, as high as 98% and for DORV, almost 99%. The rest have had an SPE of 100%. For TGA, HLH, and ToF, no false-positive diagnoses have been made. DORV has had the lowest SEN score (93.8%), whereas for TGA, HLH, ToF, and AVSD, an SEN score of 100% could be achieved. The NPV has been as high as 99% for DORV, representing the lowest score; the scores for the rest of CHD have achieved as high as 100%, reflecting the high reliability of prenatal echocardiography for these CHD. The PPV for AVSD has been 86% and was the lowest. On the contrary, DORV achieved a PPV score of 94%, and an even higher score has been achieved for TGA, HLH, and ToF, reaching 100%.

In Table 10, only the CHD with a prenatal number of individuals more than fifteen (N > 15) are demonstrated, in order to minimize a possible misleading effect on the results of smaller groups. The exceptions to these are TGA and AVSD in cardiac MRI, whose numbers in prenatal and postnatal echocardiography have been adequate. The postnatal echocardiography has achieved for different entities an SPE of 98%. It has performed for TGA, DORV, and HLH, an SPE of 99% and for TOF and AVSD, 100%. An SEN for DORV and ToF has been as high as 93%, which is lower compared to TGA, HLH, and AVSD (SEN 100%). On the whole, postnatal echocardiography has performed detection higher than 93% of the Individuals with congenital heart diseases. The postnatal echocardiography has shown an NPV higher than 98%. For DORV and ToF, an NPV has been as high as 100%. A PPV of 86% has been shown for TGA; being the lowest. A total of 14% of individuals with suspicion of TGA have had none. It is important to notice that this low PPV could be in association with the small number of individuals. The rest have shown a PPV higher than 93%. The PPV for DORV has been 94% and for HHL, 97%. In the case of ToF and AVSD, PPV has been 100%.

## 4. Discussion

Firstly, we wanted to examine agreement between fetal prenatal and postnatal echocardiography. The results show that prenatal echocardiography is a reliable diagnostic tool for conotruncal and outflow tract anomalies, left heart anomalies, and “Other anomalies”; though, with a higher rate of false-positive diagnosis for septal defects and CHD. Similarly, an additional postnatal echocardiography may be reasonable in the case of right heart anomalies, as this is indicated by the low strength of agreement and high false-negative rate in this group. Regarding the group of diagnoses TGA, DORV, HLH, ToF, and AVSD, it shows an almost perfect agreement, with 6% remaining; though, prenatally undetected and an increased risk of false-negative diagnosis for DORV, which reaches 12%. In addition, DORV shows a higher rate of false-positive diagnosis when compared to rest.

Secondly, we studied the agreement between prenatal echocardiography and postnatal MRI. The rate of prenatally undetected fetuses with CHD was, in general, low, especially for left heart anomalies, excepting right heart anomalies with a higher false-negative rate. However, right heart anomalies have demonstrated the highest PPV score in this group, indicating in this manner, a low risk of false-positive diagnosis. The highest risk of false-negative diagnosis has been shown in the case of right heart anomalies. The number of cases for MRI for TGA and AVSD has been lower than 15, and we have followed a careful interpretation of these results taking into consideration the reduced reliability of these results (Table 7). An increased rate of false-positive diagnosis has been shown for AVSD.

Thirdly, we found that the agreement in the postnatal echocardiography-postnatal MRI group has been almost perfect (Table 3 and Table 7). This proves that the echocardiography in postnatal life is a reliable tool. The complex CHD have shown the lowest strength of agreement and the highest rate of false-negative diagnosis reaching 11%.

This retrospective study leads to the conclusion that the prenatal echocardiography is a reliable diagnostic tool for both making and excluding a diagnosis of CHD. A reliable prenatal echocardiography permits an interdisciplinary approach for parental counselling. In that manner, it is possible for paediatricians and paediatric surgeons to involve, and make a plan for the delivery in, a tertiary hospital [4]. This leads to better outcomes [5,6,7]. This cohort has proven an “almost perfect” strength of agreement for all CHD in three groups. The left heart anomalies have the highest rate of strength of agreement in prenatal echocardiography and in the postnatal diagnostic methods. The second highest rate of concordance has been achieved by the group of conotruncal and outflow tract anomalies and it is in the same line with Gottliebson et al. [8]. Moreover, Gottliebson et al. have demonstrated a high detection rate for the conotruncal anomalies and for complex CHD such as univentricular heart and heterotaxy syndrome.

Regarding the group of diagnoses TGA, DORV, HLH, ToF, and AVSD, HLHS has demonstrated the highest strength of agreement in all groups (Table 7), and it is in agreement with the results of the left heart anomalies. DORV has demonstrated a high risk of increased false-negative diagnosis in prenatal echocardiography, when compared to postnatal echocardiography, and this is in line with Bensemlali et al. [9], who reported that DORV, and specifically the definition of malposition-type of great arteries, is challenging, and in 80% of cases, it can be correctly detected [9,10]; results which agree with the current study, in which in two cases with correct-detected DORV, the position of great arteries have had to be adjusted (TGA-type or Fallot-type). Regarding right heart anomalies and DORV, the high rate of false-negative diagnosis should be taken into consideration and in case of negative diagnosis, eventually a re-evaluation should be considered. The risk of false-negative diagnosis is related to the lack of re-evaluation or treatment [11]. Right heart anomalies similarly have demonstrated a higher false-negative rate in the prenatal echocardiography when this compared to postnatal MRI and in these cases, individuals are at risk of unnecessary treatments or even termination [11].

Mainly due to a high NPV, only a small number of individuals face a false-negative diagnosis. On the contrary, the risk of a false-positive diagnosis mainly for septal defects, complex CHD, and TGA, DORV, HLH, ToF, and AVSD is higher. The prenatal echocardiography has shown a low SEN score for DORV compared with rest. The false-positive rate in this group varied up to 15% for complex CHD. This may lead to unnecessary tests with consequent parents’ disquiet or even misleading decision to termination of pregnancy [12]. The CHD with a lower PPV and positive diagnosis requires, therefore, re-evaluation and adequate counselling of parents [13]. In the present study, due to the small number of individuals (N < 15) with CHD and “Other anomalies”, bias is possible. These results show common features with those of the study about precision of echocardiographic diagnosis in early pregnancy from Pike et al. [14] with an SPE of 97.3%, a PPV of 81.2%, and an NPV as high as 100%. A SEN has reached 100% in the study of Pike et al. [14], and it has been higher than in the present study (90–100%). The difference in the current study lies with the evaluation of findings of the whole pregnancy. Pike et al. [14] recommend a follow-up after 20 weeks to increase the reliability of the diagnosis. Bakiler et al. [15] could demonstrate a high SPE of 98% and predictive values higher than 90%; though, with a SEN of 42%, there is a lower reliability for detection than in the present study. In the current study, there have been generally shown higher scores than in the study of Gottliebson et al. [8]; SPE and NPV of 82–100% and SEN and PPV of 83–100%. However, it is important to take into consideration the time period of the study of Gottliebson et al., which took place from 1998 to 2003, and that it is easier to achieve better results in more recent Cohort than in older ones. Therefore, the results of the present study, that took place from 2012 to 2017 are based on better technical equipment.

The study had some limitations. Firstly, in this retrospective approach the results correspond to a collective with a CHD and they cannot be applied to general population. Our collective has been a “high risk population” that had been referred to our referral centre for final diagnosis and delivery to our maternity unit with direct connection to the paediatric cardiology centre. Ascertainment rate, therefore, was high in our study, however there are CHD cases identified prenatally born outside our centre, which have not undergone MRI examination postnatally. Individuals with milder forms of CHD that have been delivered at local hospitals have been excluded from this study because of unavailable data. Secondly, due to retrospective design of present study and focus on prenatally diagnosed CHD, this study is not able to make a reliable statement about false-negative and false-positive diagnosis. Thirdly, intrauterine demise and termination of pregnancy have been excluded from the present study. Fourthly, it is important to mention that the restricted number of Individuals in some cases could potentially lead to bias. The results in these cases should either compared with other studies with a greater number of Individuals or re-evaluated in future larger studies. Fifthly, the expertise of fetal medicine and fetal cardiology specialists in our unit has contributed to the achieved accurate results [14,16,17].

## 5. Conclusions

The careful observation and evaluation of the results of the current study leads to the conclusion that the prenatal echocardiography represents a reliable method of detection and exclusion of CHD. It is important to mention that the experience of the examiner and eventually a follow-up to adjust the diagnosis in some cases are essential. The greatest reliability could be shown for the anomalies of the left heart and specifically for hypoplastic left heart in the present study. The anomalies of the right heart, on the contrary, have shown the highest rate of false-negative diagnosis. Septal defects have possessed the highest rate of false-positive diagnosis. The greatest challenge for an accurate diagnosis has been related to DORV due to its extreme anatomical variability and frequent association with complex abnormalities. This study shows that cardiac MRI is an additional diagnostic tool for the detailed study of the vascular anatomy and pulmonary supply. In general, it is recommended to re-evaluate the results of the present study in future studies with “low risk collective” and examiners with varying experience. Further studies with a greater number of individuals are essential for comparing and verifying our results. Additionally, it would be interesting to assess in future studies the concordance in relation to gestational week and evaluate the best time to detect progressive CHD. Lastly in case of a prospective study it would be meaningful to include false-positive and false-negative results.

## Figures and Tables

**Table 1:** Classification of congenital heart defects in current study.

Congenital Heart Disease	Diagnosis
Conotruncal and outflow tract anomalies	Transposition of great arteries (TGA) Double outlet left ventricle (DOLV) Double outlet right ventricle (DORV) Truncus arteriosus communis (TAC)
Anomalies of left heart	Aortic valve stenosis Hypoplastic left heart (HLH) Interruption of the aortic arch—aortic arch hypoplasia
Anomalies of right heart	Insufficiency of the tricuspid valve Atresia of the tricuspid valve Pulmonary valve stenosis Atresia of pulmonary valve (PAT) Absent pulmonary valve Ebstein anomaly Tetralogy of Fallot (TOF)
Septal defects	Ventricular septal defect (VSD) Atrial septal defect (ASD) Atrioventricular septal defect (AVSD)
Complex CHD	Univentricular heart (UVH) Isomerism: L-isomerism, R-isomerism
Other anomalies	Ectopia cordis cardiac tumours

**Table 2:** Frequency of congenital heart defects in present collective.

	Prenatal Echocardiography (%)	Postnatal Echocardiography (%)
Conotruncal/anomalies of outflow tract	*21.4*	*21.5*
Transposition of great arteries	9	9.6
Double outlet left ventricle	0.6	0.6
Double outlet right ventricle	10.7	10.2
Anomalies of left heart	*26.6*	*28.3*
Stenosis of the aortic valve	3.4	4
Hypoplastic left heart	19.8	20.3
Interrupted aortic arch	0.6	0.6
Hypolastic aortic arch	2.8	3.4
Anomalies of right heart	*21*	*22.7*
Insufficiency of tricuspid valve	0.6	1.1
Atresia of tricuspid valve	1.7	2.3
Stenosis of pulmonary valve	2.3	2.3
Pulmonary atresia	1.7	2.3
Absent pulmonary valve	1.1	1.1
Ebstein anomaly	0.6	0.6
Tetralogy of Fallot	13	13
Septal defects	*19.2*	*17*
Atrial septal defect	0	0.6
Ventricular septal defect	3.4	2.8
Atrioventricular septal defect	15.8	13.6
Complex CHD	7.9	6.8
Univentricular heart	6.2	5.1
L-isomerismus	1.1	1.1
R-isomerismus	0.6	0.6
Other anomalies	5.1	5.1
Ectopia cordis	1.1	1.1
Tumours	1.1	1.1
Number of patients	177	177

**Table 3:** Strength of agreement; comparison among prenatal echocardiography, postnatal echocardiography and cardiac MRI.

Diagnosis	Concordance (Cohen’s Kappa)		
	Prenatal-Postnatal Echocardiography	Prenatal Echocardiography-MRI	Postnatal Echocardiography-MRI
Conotruncal and outflow tract anomalies	0.933	0.946	0.974
Anomalies of left heart	0.957	0.98	0.98
Anomalies of right heart	0.917	0.941	0.971
Septal defects	0.924	0.913	1
Complex defects	0.917	0.942	0.936
Other anomalies	1	1	1

**Table 4:** Sensitivity (SEN), specificity (SPE), positive (PPV) and negative predictive value (NPV) for diagnosis in the prenatal echocardiography. Reference: The postnatal echocardiography. N = 177.

	Prenatal Echocardiography				
	Prevalence of Prenatal Echocardiography (%)	SEN (%)	SPE (%)	PPV (%)	NPV (%)
Conotruncal and outflow tract anomalies	21.5	94.7	98.6	94.7	98.6
Anomalies of left heart	26.6	94	100	100	97.7
Anomalies of right heart	20.9	90	99.3	97.3	97.1
Septal defects	19.2	100	97.3	88.2	100
Complex congenital heart defects	8.5	100	98.8	85.7	100
Other anomalies	5.1	100	100	100	100

**Table 5:** Sensitivity (SEN), specificity (SPE), positive (PPV) and negative predictive value (NPV) for diagnosis in the prenatal echocardiography. Reference: Postnatal cardiac. MRI: 108.

	Prenatal Echocardiography				
	Prevalence Prenatal (%)	SEN (%)	SPE (%)	PPV (%)	NPV (%)
Conotruncal and outflow tract anomalies	22.2	95.8	98.8	95.8	98.8
Left heart anomalies	36.1	97.5	100	100	98.6
Right heart anomalies	18.1	90.9	100	100	97.7
Septal defects	13	100	97.9	85.7	100
Complex congenital heart defects	9.3	100	99	90	100
Other anomalies	2.8	100	100	100	100

**Table 6:** Sensitivity, specificity, positive and negative predictive value for the diagnosis in postnatal echocardiography. Reference: Diagnosis in cardiac MRI. N = 108.

	Postnatal Echocardiography and MRI				
	Prevalence Postnatal (%)	SEN (%)	SPE (%)	PPV (%)	NPV (%)
Conotruncal and outflow tract anomalies	23.1	100	98.8	96	100
Anomalies of left heart	38	100	98.5	97.6	100
Anomalies of right heart	19.4	95.5	100	100	98.9
Septal defects	11.1	100	100	100	100
Complex CHD	7.4	88.9	100	100	99
Other anomalies	2.8	100	100	100	100

**Table 7:** Strength of agreement; comparison among prenatal echocardiography, postnatal echocardiography and cardiac MRI.

	Concordance (Cohen’s Kappa)		
	Prenatal and Postnatal Echocardiography	Prenatal Echocardiography and MRI	Postnatal Echocardiography and MRI
Transposition of great arteries	0.967	1	0.918
Double outlet right ventricle	0.849	0.927	0.927
Hypoplastic left heart	0.982	1	0.976
Tetralogy of Fallot	0.950	1	0.960
Atrioventricular septal defect	0.910	0.913	1

**Table 8:** Sensitivity, specificity, positive (PPV) and negative predictive value (NPV) for the diagnosis in the prenatal echocardiography. Reference: Diagnosis in the postnatal echocardiography. N = 177.

	Prenatal Echocardiography				
	Prevalence (%)	SEN (%)	SPE (%)	PPV (%)	NPV (%)
Transposition of great arteries	9	94.1	100	100	99.4
Double outlet right ventricle	10.7	88.9	98.1	84.2	98.7
Hypoplastic left heart	19.8	97.2	100	100	99.3
Tetralogy of Fallot	13	95.7	99.4	95.7	99.4
Atrioventricular septal defect	15.8	100	97.4	85.7	100

**Table 9:** Sensitivity, specificity, positive and negative predictive value for diagnosis in the prenatal echocardiography. Reference: Diagnosis in cardiac MRI. N = 108.

	Prenatal Echocardiography				
	Prevalence Prenatal (%)	SEN (%)	SPE (%)	PPV (%)	NPV (%)
Transposition of great arteries	5.6	100	100	100	100
Double outlet right ventricle	14.8	93.8	98.9	93.8	98.9
Hypoplastic left heart	26.9	100	100	100	100
Tetralogy of Fallot	13.9	100	100	100	100
Atrioventricular septal defect	13	100	97.9	85.7	100

**Table 10:** Sensitivity, specificity, positive and negative predictive value in the postnatal echocardiography. Reference: Diagnosis in cardiac MRI. N = 108.

	Postnatal Echocardiography and MRI				
	Prevalence Postnatal (%)	SEN (%)	SPE (%)	PPV (%)	NPV (%)
Transposition of Great arteries	6.5	100	99	85.7	100
Double outlet right ventricle	14.8	93.8	98.9	93.8	98.9
Hypoplastic left heart	26.9	100	98.8	96.6	100
Tetralogy of Fallot	13	93.3	100	100	98.9
Atrioventricular septal defect	11.1	100	100	100	100

## Data Availability

Data is unavailable due to privacy or ethical restrictions.
